# 

**Published:** 1918-03-23

**Authors:** 


### CONTENTS.

| | PAGE |
|---|---|
| **Reports of Scottish Asylums** ... ... ... | 517 |
| **Organisation: Voluntary, or Compelled?** ... | 518 |
| **Hospital and Institutional News** ... ... ... | 519 |
| Annual Meetings as Money-Spinners—Brain-workers and their Food—A New Financial Policy at Cardiff—Democracy and Management—The End of Inaction at Devonport—Begging in the Wards—The Waiting List at Nottingham—The Development of Workpeople's Contributions—The Queen and the Chelsea Hospital for Women—Three Gifts to Queen's Hospital, Birmingham—The New President of the Royal Lancaster Infirmary—Mr. Robey's Presentation—Sectional Subscriptions in Cathedral Towns—Hahnemann and Public Confidence—The Prince of Wales' Inspiration—Sailors' and Soldiers' Dependents in Rate-aided Institutions—Tetanus Germs in Catgut—War Prices at Rhyl—Through the Eyes of a Child—A Dispenser's Record of Service. | |
| **The Great War** ... ... ... ... ... ... | 523 |
| IV. Sickness. | |
| **Lord Lister** ... ... ... ... ... ... | 525 |
| IX. The Drainage Tube, and Ligatures. | |
| **The Finance of Surgery** ... ... ... ... | 527 |
| A Study of the "End Result" System. | |
| **The Muse of Hospital History** ... ... ... | 530 |
| Mr. D'Arcy Power's Evokement of "Bart.'s." | |
| **The Material Value of Æsthetics** ... ... | 529 |
| **The Navy and Our Sailors** ... ... ... ... | 531 |
| A Memorable Annual Meeting. | |
| **Parliament and the Profession** ... ... ... | 532 |
| **The Matrons' and Sisters' Department** ... | 533 |
| Nursing Progress and its Developments: X. Local Centres for Examinations. | |
| Round the Hospitals. | |
| Army Nurses' Grievances—Dancing at the Front—Are Examination Results Always Correct?—The College and the Smaller Training-schools—Army Nurses' Relatives and Pensions—Ration Difficulties in Institutions? | |
| **Nursing Affairs in Ireland** ... ... ... ... | 536 |
| Toward Union. | |
| **The Institution Garden** ... ... ... ... | 537 |
| II. What to Do and How to Do it. | |
| **Matrons' and Sisters' Appointments** ... ... | 538 |
| **The British Hospitals Association** ... ... | 539 |
| Payment of the Medical Staff. | |
| **The Institutional Worker** ... ... | (Suppl.) 1 |
| How to Sterilise Rubber Gloves: Answer to February Question. | |
| Hot Dinner-plates: Answer to February Question. | |
| March Questions. | |
| Questions Invited. | |

